# High-fidelity optical diffraction tomography of multiple scattering samples

**DOI:** 10.1038/s41377-019-0195-1

**Published:** 2019-09-11

**Authors:** Joowon Lim, Ahmed B. Ayoub, Elizabeth E. Antoine, Demetri Psaltis

**Affiliations:** Ecole Polytechnique Fédérale de Lausanne, Optics Laboratory, CH-1015 Lausanne, Switzerland

**Keywords:** Optical physics, Optical physics

## Abstract

We propose an iterative reconstruction scheme for optical diffraction tomography that exploits the split-step non-paraxial (SSNP) method as the forward model in a learning tomography scheme. Compared with the beam propagation method (BPM) previously used in learning tomography (LT-BPM), the improved accuracy of SSNP maximizes the information retrieved from measurements, relying less on prior assumptions about the sample. A rigorous evaluation of learning tomography based on SSNP (LT-SSNP) using both synthetic and experimental measurements confirms its superior performance compared with that of the LT-BPM. Benefiting from the accuracy of SSNP, LT-SSNP can clearly resolve structures that are highly distorted in the LT-BPM. A serious limitation for quantifying the reconstruction accuracy for biological samples is that the ground truth is unknown. To overcome this limitation, we describe a novel method that allows us to compare the performances of different reconstruction schemes by using the discrete dipole approximation to generate synthetic measurements. Finally, we explore the capacity of learning approaches to enable data compression by reducing the number of scanning angles, which is of particular interest in minimizing the measurement time.

## Optical Diffraction Tomography: Seeing things in better light

The resolution of an imaging technique called optical diffraction tomography (ODT) is improved using a novel software algorithm and learning procedure. ODT is similar to the computerised tomography process of medical CT scanning, or CAT scanning, but using light rather than X-rays. A sample is illuminated from various angles and the phase and intensity of the diffracted light is analysed and processed to generate images of the sample’s fine details. Joowon Lim and colleagues led by Demetri Psaltis at the Swiss Federal Institute of Technology in Lausanne refined the technique to produce what they call a “high fidelity” version by using a more sophisticated method for analysing the light beams. The technique is especially useful for imaging complex biological samples such as tissue slices and living cells. Imaging yeast cells demonstrates the increased power that is achieved.

## Introduction

Quantitative-phase imaging (QPI) enables the measurement of the phase-contrast information of transparent samples such as biological cells. QPI contrast is generated from the refractive index (RI) contrasts within and around a sample. Because this contrast mechanism is endogenous, quantitative-phase information does not require external labeling, such as immunostaining, which may perturb the sample. QPI contains the coupled information of sample thickness and RI contrast. Optical diffraction tomography (ODT) provides the 3D RI distribution of a sample by combining multiple 2D QPI measurements from various illumination angles^1,2^. Reconstructed tomograms provide structural information that has been extensively utilized to study hematology^3,4^, morphological parameters^5^, and biochemical information^6^ which are summarized in several review papers^2,7–9^. In ODT, the way in which multiple 2D measurements are combined into unified 3D information is critical. Under the assumption of a weakly scattering sample, the Wolf transform^10^ has been widely used. Depending on how the 2D projections are processed, we obtain either the Born or Rytov approximations for the Wolf transform^1^. Each method has its limitations^11^, but the Rytov approximation is known to be more appropriate than the Born approximation for many biological applications^12^. However, when a sample is thicker and more complex, the Rytov approximation is no longer valid. This limits the usefulness of ODT for imaging complex samples.

Recently, methods have emerged to overcome the limitations of the Born and Rytov approximations by taking multiple scattering into account^13–20^. It was shown using Mie theory^20^ that learning tomography (LT)^14,21^, an approach that exploits the beam propagation method (BPM) as the forward model to capture multiple scattering, has superior performance compared with that of the conventional imaging method based on the Rytov approximation. We refer to it as LT-BPM. LT uses the forward model of dividing 3D samples into multislices followed by slice-by-slice propagations. Due to the multislice modeling of forward models by LT, the resulting structure is similar to that of a neural network, and we can use the error back-propagation algorithm to calculate the gradient. The BPM consists of two steps: non-paraxial diffraction followed by phase modulation. The diffraction step used in the BPM assumes $k_0n\left( {x,y,z} \right) \approx k_0n_0$, where *k*_0_ is the free-space wavenumber, *n*_0_ is the RI of the medium, and *n*(*x,y,z*) represents RI variations. In addition, the phase modulation steps use a distance, *dz*/cos*θ*, to modulate the phase throughout propagation, given the propagation step (*dz*) and the illumination angle (*θ*). However, for thicker and more complex samples, as light propagates through the samples, multiple diffracted beams of light are generated, and it is not valid to use one single value, *dz*/cos*θ*, to represent optical path lengths. This deviation from the fixed distance, *dz*/*cosθ*, increases with increasing the illumination angle due to the nature of the cosine function^22^.

In this paper, we show that the accuracy of LT reconstructions of a 3D object is increased when we use the split-step non-paraxial (SSNP) method rather than the BPM. We refer to it as LT-SSNP. The SSNP method exploits not only the field but also the derivative of the field along the optical axis to model the propagation^23,24^. While the BPM requires this approximation,$k_0n\left( {x,y,z} \right) \approx k_0n_0$, to decouple diffraction from phase modulation, SSNP does not require the approximation, benefiting from propagating the derivative of the field at the same time. Phase modulation affects the derivative and is used concurrently in the next step of the diffraction calculation. LT-SSNP uses the same iterative scheme used in LT-BPM. To fairly assess LT-SSNP and compare it with the LT-BPM, synthetic measurements are generated using Mie theory and the discrete dipole approximation (DDA). For spherical and cylindrical objects, Mie theory provides the analytical solution to the Helmholtz equation^25^. Therefore, the solution of Mie theory takes into account multiple scattering. Here, we also use the DDA to simulate light scattering by an arbitrarily shaped sample to generate more complex synthetic data. The DDA is a general method for calculating the scattering and absorption caused by an arbitrarily shaped sample represented by finite discrete dipoles^26^. These dipoles react not only to incident light but also to one another, which places the resulting fields under high orders of scattering. It has been shown that the DDA works well for samples whose RI values fairly match those of the surroundings, such as biological cells in a liquid medium^27^. Therefore, we use Mie theory for multiple cylinders and the DDA for a cell phantom, as well as a cluster of 15 red blood cells (RBCs). After generating synthetic measurements by using either Mie theory or the DDA, the LT-BPM and LT-SSNP are used to reconstruct the 3D RI of each sample, and the accuracy of each reconstruction is evaluated quantitatively.

In this analysis, we include an investigation of the performance of each algorithm with respect to regularization. The iterative reconstruction scheme used for both the LT-BPM and LT-SSNP minimizes a cost function that comprises two terms: data fidelity and regularization. The data fidelity term is defined by whether the forward model applies either the BPM or the SSNP, and the regularization term introduces prior knowledge about the sample characteristics such as edge sparsity and non-negativity. The relative importance of the two terms in the cost function is controlled by the regularization parameter. We compare the LT-BPM and LT-SSNP by using varying regularization parameters with the goal of minimizing the influence of the regularization term so that the results are primarily based on the forward model rather than on prior knowledge. For the simulations described, we confirm that LT-SSNP shows lower dependency on the regularization parameter due to the accuracy of SSNP. In other words, the use of a more accurate forward model permits LT-SSNP to extract more information from the measurements and to rely less on regularization. More importantly, for highly aggregated samples subject to significant multiple scattering, LT-SSNP allows individual objects and structures to be clearly distinguished, while this observation cannot be made when using the LT-BPM.

We validate the proposed method by using experimental ODT data from a yeast cell and from HCT116 human colon cancer cells. To image biological cells with fine details, it is critical to reduce the influence of the regularization term, as high regularization not only smooths out the imaging artifacts but also useful information, leading to deterioration in the quality of the reconstruction. Tomograms of a yeast cell reconstructed by using LT-SSNP show successful results with high quality even with a very low regularization parameter, while the LT-BPM fails to recover fine details within and around the cells. In the case of experimental measurements of biological cells, the true RI distribution is not known, which prevents the direct assessment of the accuracy of the various ODT methods. To overcome this issue, we generate two sets of semisynthetic measurements by using the DDA for each of the RI reconstructions from the LT-BPM and LT-SSNP. A comparison of the discrepancies between the semisynthetic and experimental measurements reflects the proximity of each solution to the real RI values.

Finally, we explore the capacity of LT-SSNP to produce accurate reconstructions with a reduced number of illumination angles^28,29^. This is of particular interest because the number of scanning angles is directly related to the measurement time. A comparison of each reconstruction method for a varying number of scanning angles indicates that learning approaches provide a dramatic improvement over conventional methods. Overall, the more accurate forward model used in LT-SSNP translates to excellent results even with low regularization and a small number of illumination angles.

## Results

In this section, we compare the LT-BPM and LT-SSNP, which belong to the same family of LT reconstruction schemes, except for the forward models, namely, the BPM and the SSNP, respectively. LT minimizes the cost function, which consists of two terms as follows:

1 $$\widehat {\bf{x}} = \arg \min _{{\bf{x}} \in P}\frac{1}{{2L}}\mathop {\sum}\limits_{l = 1}^{L} | |{\mathbf{y}}_{K}^{(l)} - {\mathbf{S}}_{K}^{(l)}({\bf{x}})||_{2}^{2} + \tau R({\bf{x}})$$

where the first term is the data fidelity term and *R* is the 3D total variation (TV)^30^ regularization term to impose edge sparsity on the solution. The relative importance between two terms is controlled by the regularization parameter, *τ*. ${\mathbf{y}}_K^{\left( l \right)} \in {\Bbb C}^M$ denotes the experimental measurements at the *K*th slice for each illumination angle *l*, and *L* is the total number of angles. ${\mathbf{S}}_K^{\left( l \right)}\left( {\mathbf{x}} \right)$ represents the estimate by a forward model (either the BPM or the SSNP) at the *K*th slice, which is the last slice of the volume, to be compared with ${\mathbf{y}}_K^{\left( l \right)}$ given a current solution, ${\mathbf{x}} \in {\Bbb R}^N$. $P \in {\Bbb R}^N$ is a convex set that imposes a non-negativity constraint. In the supplementary section, we describe the calculation of the gradient for SSNP. Once we calculate the gradient of the data fidelity term in Eq. (1), the optimization scheme uses the fast iterative shrinkage-thresholding algorithm (FISTA)^31^ as explained in ref. ^21^ for 3D isotropic TV regularization, with eight randomly chosen angles in each iteration.

### Multiple cylinders by using Mie theory

**Fig. 1:**
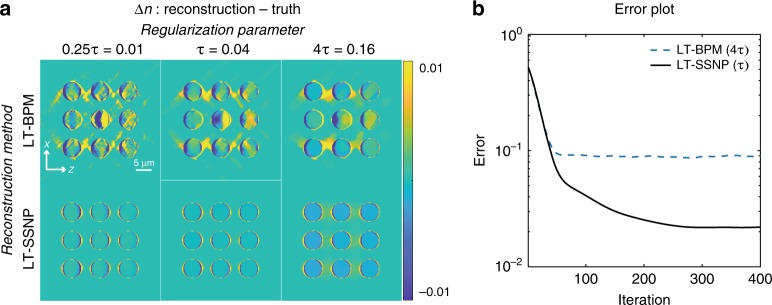
Reconstruction results of cylinders using the LT-BPM and LT-SSNP for various regularization parameters (*τ* = 0.04). **a** Difference maps between reconstructions from the LT-BPM/LT-SSNP and the ground truth (reconstruction—truth). **b** Plots of the *Error* of the LT-BPM and LT-SSNP by using the regularization parameter that produced the minimum *Error* value

We applied the LT-BPM and LT-SSNP on a highly scattering simulated sample consisting of a 3 × 3 grid of cylinders. Each cylinder is 6 μm in diameter with an RI of 1.05 immersed in air. The center-to-center distance is 9 μm. We varied the regularization parameter to investigate the accuracy of the forward model for each algorithm. The results are presented by mapping the difference between the reconstructed tomogram for each method and the known solution, as shown in Fig. 1a. The LT-BPM shows many artifacts inside the cylinders and smearing of the RI in the region between the cylinders. These artifacts of the forward model cannot be eliminated even by increasing the regularization parameter. Regularization only smooths out the overall reconstruction. In contrast, LT-SSNP clearly distinguishes each cylinder without interstitial artifacts even with the weakest regularization parameter tested, that is, 0.25*τ* = 0.01. Interestingly, increasing the regularization parameter to 4*τ* = 0.16 reduces the reconstruction quality when using the LT-SSNP algorithm. The total *Error*, which is defined as follows:

2 $$Error\,({\bf{x}}_{\mathrm{recon}},{\bf{x}}_{\mathrm{true}}) = \frac{{||{\bf{x}}_{\mathrm{recon}} - {\bf{x}}_{\mathrm{true}}||^2}}{{||{\bf{x}}_{\mathrm{true}}||^2}}$$

was also calculated as a function of the iteration number, as shown in Fig. 1b. **x**_recon_ is the reconstructed RI contrast from the medium RI, and **x**_true_ is the ground truth RI contrast. Figure 1b displays the *Error* plots of the LT-BPM and LT-SSNP by using the regularization parameter that produced the lowest *Error* value for each algorithm: 4*τ* = 0.16 for the LT-BPM and *τ* = 0.04 for LT-SSNP. This analysis quantitatively confirms the better accuracy of LT-SSNP. In the case of multiple cylinders, it is critical to model distortions in the wavefront (phase modulation) introduced by the precedent samples, which determine the illumination on subsequent samples. We further analyzed this scenario by varying the number of layers in multiple cylinders and summarized the results in the supplementary material.

### RBC cluster using discrete dipole approximation

**Fig. 2:**
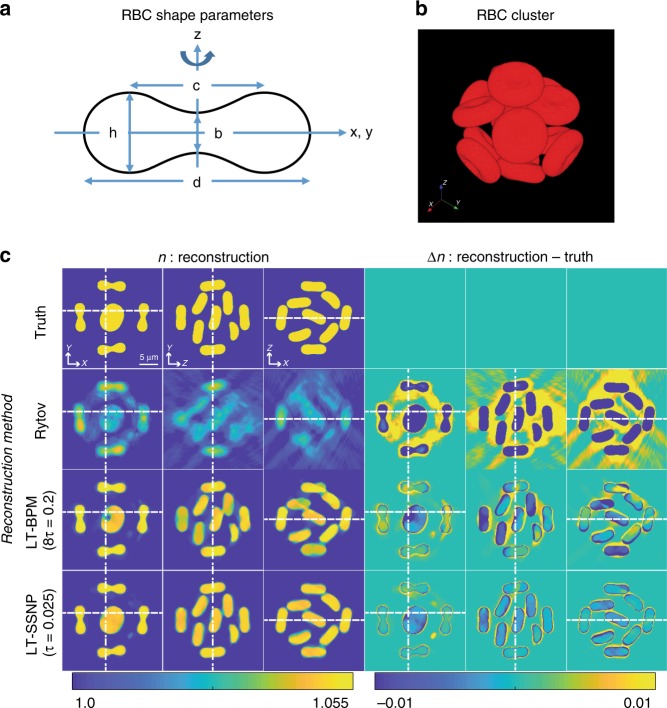
Reconstruction results of a RBC cluster using the LT-BPM and LT-SSNP. **a** Parameters used to define the shape of the RBC. **b** 3D rendering of the RBC cluster, which consists of 15 identical RBCs. **c** 3D RI and difference maps for a cluster of 15 RBCs. Top to bottom: ground truth, Rytov approximation, LT-BPM, and LT-SSNP (*τ* = 0.025). Left: *xy*, *yz*, and *xy* slices of the 3D RI. Right: difference maps between the reconstructed RI and the ground truth

**Fig. 3:**
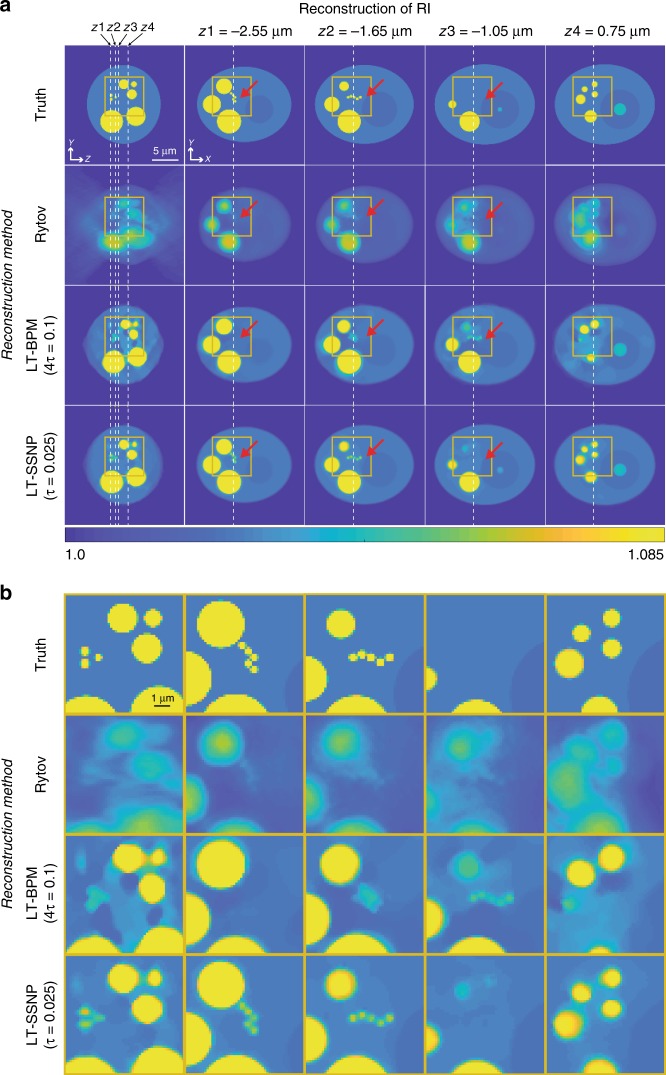
Reconstruction results of a cell phantom by using Rytov, the LT-BPM, and LT-SSNP at four different *z* planes (*τ* = 0.025). **a** Left column: the *yz* slice, with the *z*-positions of the *yx* slices indicated by using dashed lines. Second through fifth columns: *yx* slices at the positions indicated in the left column. **b** Magnification of the regions indicated by the boxes in part (**a**)

To investigate the performance of each algorithm with highly scattering samples in 3D, we performed a similar test on a simulated cluster of RBCs. The shape of a single RBC is sketched in Fig. 2a, while the organization of the cluster is shown in Fig. 2b. Reconstructions were performed by using various regularization parameters; for each algorithm, we show only the reconstruction by using the regularization parameter that gives the lowest *Error*: 8*τ* = 0.2 for the LT-BPM and *τ* = 0.025 for LT-SSNP. In Figs. 2c, 3, different slices (*xy*, *yz*, and *xz*) of the 3D RI distributions resulting from each method are presented, along with the difference map with respect to the ground truth. Both the LT-BPM and LT-SSNP show better reconstructions compared with reconstructions based on the Rytov approximation, which is expected since Rytov does not consider multiple scattering. By comparing the LT-BPM and LT-SSNP, we can see that the RI tomogram resulting from LT-SSNP shows clearer and more accurate reconstructions of each RBC, producing homogeneous RI distributions within each RBC.

### Cell phantom using discrete dipole approximation

To evaluate the LT-BPM and LT-SSNP algorithms on a sample whose RI values are not homogeneous and which contains fine details, we generated a synthetic cell phantom. The phantom contains four different RI values corresponding to the cytoplasm, nucleus, nucleolus, and lipids^32^. Synthetic measurements were made by using the DDA in the same manner as for the RBCs. Again, we present for each algorithm only the results obtained by using the regularization parameter that produced the lowest *Error* value (4*τ* = 0.1 for the LT-BPM and *τ* = 0.025 for LT-SSNP). The reconstruction results are shown in Fig. 3a. Figure 3b displays the magnified regions, which are indicated by the yellow boxes in Fig. 3a. The reconstructions obtained with the LT-BPM show that the cytoplasmic regions are highly distorted, similar to the artifacts observed inside the cylinders and RBCs. More importantly, the small lipids indicated by the red arrows are hardly distinguishable due to the inaccuracy of the BPM. By contrast, the LT-SSNP not only distinguishes the shapes of fine structures but also correctly positions them along the optical axis.

### Experimental validation using a yeast cell

**Fig. 4:**
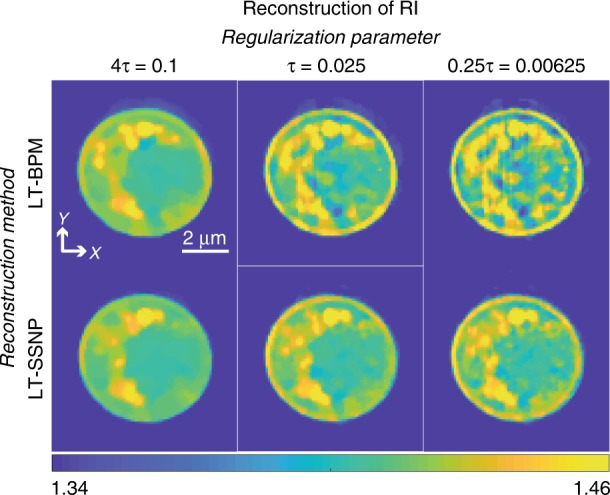
Reconstruction results of a yeast cell by using the LT-BPM and LT-SSNP for various regularization parameters (*τ* = 0.025)

**Fig. 5:**
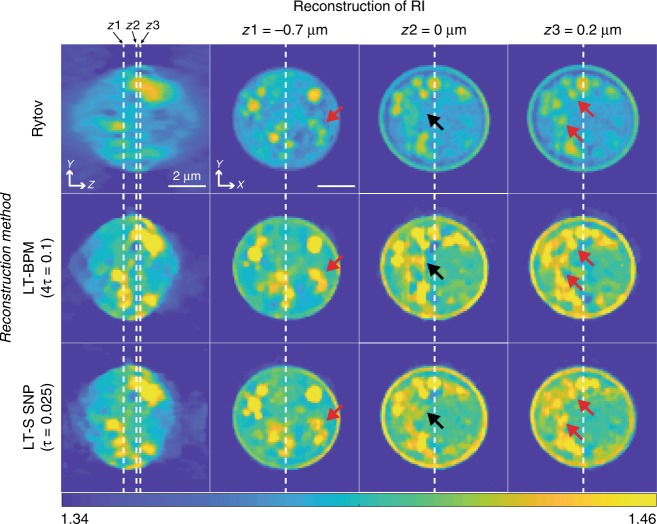
Reconstruction results of a yeast cell by using Rytov, the LT-BPM, and LT-SSNP at three different *z* planes (*τ* = 0.025)

To validate the relative performances of the LT-BPM and LT-SSNP on experimental data, we acquired ODT images of a yeast cell. Again, we evaluated different regularization parameters for the reconstructions obtained by using the LT-BPM and LT-SSNP. Figure 4 shows the reconstruction results for a slice close to the image plane. For both the LT-BPM and LT-SSNP, the high regularization parameter 4*τ* = 0.1 results in too much smoothing, and it becomes difficult to resolve fine details. Therefore, it is necessary to reduce the regularization parameter. However, in the case of the LT-BPM, lowering the value of the regularization parameter introduces artifacts similar to those present in the simulation results in the previous section. By contrast, the LT-SSNP can reconstruct fine details without introducing strong artifacts. Therefore, we used *τ* = 0.025 for the LT-BPM and *τ/*4 = 0.00625 for LT-SSNP and further analyzed the sample for different z planes, as shown in Fig. 5. Since we used higher regularization for the LT-BPM, we can clearly see that images tend to be smoothed out and fine details are lost, as indicated by the red arrows in Fig. 5. By contrast, the LT-SSNP reveals structures that are not observable in the Rytov and LT-BPM reconstructions. In addition, even with the higher regularization, the LT-BPM still shows several artifacts, as indicated by the black arrows in Fig. 5.

**Fig. 6:**
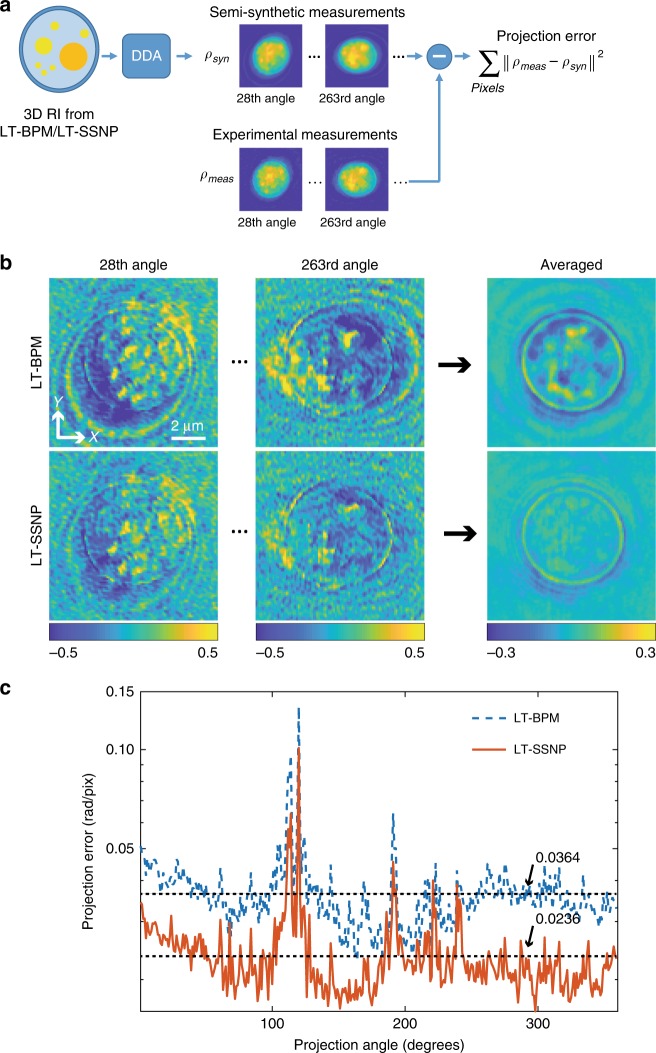
Evaluation of LT algorithms by using semisynthetic error estimation. **a** Overall scheme of semisynthetic measurement generation by using the DDA. **b** Phase-difference maps for two randomly selected angles and the average for all angles. The color bar is in radians. **c** Calculation of the projection error in retrieved-phase information from experimental measurements and semisynthetic data

A serious limitation for quantification of the reconstruction accuracy for real biological samples such as this yeast cell is the fact that the true RI distribution is unknown. However, we were able to further evaluate the differences between the LT-BPM and the LT-SSNP by using the semisynthetic measurements generated by using the DDA. While we generated synthetic measurements by using synthetic samples in all previous cases, the RI reconstructions obtained by using the LT-BPM and the LT-SSNP served as samples for the DDA to generate semisynthetic measurements in this case. The projection error—the difference in phase information between the experimental data and these simulated measurements—reflects how close the solution is to the true RI distributions, as shown in Fig. 6a. Figure 6b maps the 2D projection error for two randomly selected angles as well as the average across the full set of angles for each algorithm. In the case of the LT-BPM, differences are clearly observed when compared with the LT-SSNP, which shows remarkable consistency with the experimental measurements. We quantified the mean projection error (radians/pixel) for each and used this metric to quantify the accuracy of the LT-SSNP compared with that of the LT-BPM (Fig. 6c). The average projection error across all angles was 65% lower for the LT-SSNP than for the LT-BPM.

### Data compression demonstrated on experimental data—HCT116 cells

**Fig. 7:**
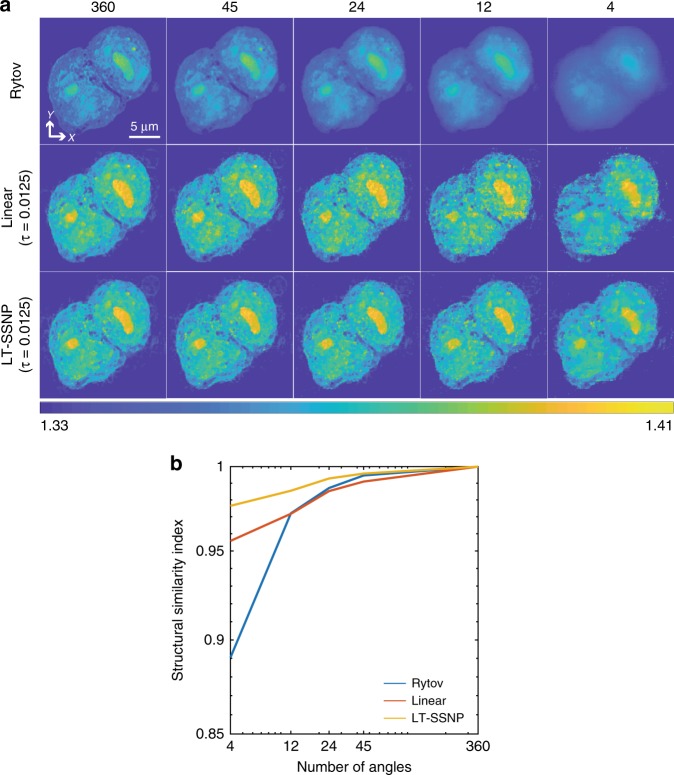
Data compression demonstrated on HCT116 cells. **a** Reconstruction results of HCT116 human colon cancer cells by using Rytov, linear tomography, and the LT-SSNP for a downsampled number of scanning angles. **b** Structural similarity index plot with respect to the reconstruction by using each method with full measurements

The tomographic reconstruction based on the Wolf transform and the Rytov approximation directly maps multiple 2D measurements into the 3D Fourier space. Therefore, any missing information in measurements directly deteriorates the final reconstruction. However, the LT-SSNP is an iterative reconstruction scheme. The iterative reconstruction begins with an initial guess (usually based on the Rytov approximation), and the initial solution is updated based on the calculated error gradient by using the forward model. In addition, prior knowledge about the sample is imposed on the current guess during the iterative process. Therefore, even if the measurements are underdetermined due to missing measurements, the learning approaches can fill in some of the missing information. This idea was validated by reducing the number of illumination angles used for each method. The experimental data used for this investigation were ODT images of a pair of HCT116 human colon cancer cells. These cancerous epithelial cells contain information in small structures relative to the size of the cell and highlight the importance of reconstructions that can capture these fine details. Reconstructions were performed by using Rytov, linear tomography^20^, and the LT-SSNP by using different numbers of projection angles (45, 24, 12, and 4) uniformly spaced in the range from 0 to 360°. The linear tomography method uses the same iterative reconstruction scheme as the LT-SSNP, except with single scattering as the forward model. For the quantitative analysis, we also compare the structural similarity index (SSIM)^33^ for reconstructions from compressed measurements with the full measurement case, namely, 360 angles at the focal plane. The results, plotted in Fig. 7, show a dramatic improvement in the reconstruction quality for linear tomography and the LT-SSNP because the two methods iteratively fill up empty components introduced from missing measurements but using different forward models. In the case of the HCT116 cells, Rytov produces fairly good reconstructions that reveal intracellular structures with 360 full projections, despite the underestimation due to the missing-cone problem. The Rytov reconstructions, on the other hand, rapidly deteriorate as the number of illumination angles decreases. Compared with Rytov and linear tomography, the LT-SSNP is more robust in the number of projections, providing reconstructions with only four scanning angles with nearly the same quality as reconstructions by using the full 360-angle data, as confirmed by the SSIM in Fig. 7b. We believe that the LT-SSNP can benefit from both the iterative scheme and an accurate forward model. In addition, we further tested the compression using the cell phantom, which has higher RI contrasts; the results have been added to the supplementary material.

## Discussion

In this study, we have proposed a new tomographic reconstruction algorithm, the LT-SSNP, which is based on the SSNP forward model, for imaging complex highly scattering samples with fine details. By benefiting from the accuracy of the SSNP, the LT-SSNP extracts a maximum amount of information from measurements rather than relying on prior assumptions and generalizations about the sample structure. The LT-SSNP was quantitatively evaluated and compared with the previous algorithm, the LT-BMP, by using synthetic measurements. These synthetic measurements with a known solution were generated by using Mie theory for multiple cylinders, and the DDA for an arbitrarily shaped cluster of RBCs and a cell phantom.

In the case of multiple cylinders, the LT-SSNP shows clear reconstruction of each sample without introducing artifacts. The more interesting point is that the LT-SSNP does not require strong regularization. This is because the SSNP forward model is accurate enough that regularization is not necessary to compensate for poor data fidelity, while the LT-BPM could not properly carry out the reconstruction even with high regularization. For the RBC cluster in 3D, the LT-SSNP returns more homogeneous distributions even with a lower value of the regularization parameter than that of the LT-BPM. This fact is critical when imaging complex samples because too much regularization smooths out fine structures and makes them impossible to resolve. The cell phantom simulation confirms the performance of the LT-SSNP on a sample with high-resolution information. The LT-SSNP is more accurate and permits the use of a lower regularization parameter, which allows details of the 3D refractive index to be identified without artificially being smoothed out by regularization.

Importantly, the added capabilities of the LT-SSNP are dramatic for imaging biological samples containing information across many scales, as confirmed by applying it to tomographic images of a yeast cell. The reconstructed tomograms by using the LT-SSNP clearly reveal structures that are not observable in the case of Rytov and the LT-BPM. Semisynthetic measurements based on the RI reconstructions of the LT-BPM and the LT-SSNP numerically validate the accuracy of the LT-SSNP reconstructions. The averaged phase-difference map represents how close the reconstruction using each method is to the real sample. In contrast to the averaged phase-difference map of the LT-BPM, which produces many discrepancies inside the sample, that of the LT-SSNP shows consistency with the experimental measurements. The numerical evaluation shows that the LT-SSNP produces a 65% reduction in the projection error compared with that of the LT-BPM.

Furthermore, we explored the capacity of learning approaches to enable data compression by reducing the number of scanning angles. The LT-SSNP shows a dramatic improvement in image quality by using a small number of illumination angles when compared with the conventional direct inverse method by using the Rytov approximation. Even with a low number of projections, the LT-SSNP benefits from its weak dependency on the regularization parameter.

## Materials and methods

### Simulation

We used Mie theory to derive the field scattered by multiple cylinders (2D)^34^. A total of 101 illumination angles uniformly distributed between −45° and 45° were used. To perform a deeper assessment of the LT-BPM and LT-SSNP algorithms, we also tested on synthetic measurements in arbitrary-shaped samples: an RBC cluster and a cell phantom.

For RBC simulations, the discrete dipole approximation^26,35^ was applied to an RBC cluster, in which the surface of each RBC is defined by using the following equation:

3 $$\rho ^4 + 2S\rho ^2z^2 + z^4 + P\rho ^2 + Qz^2 + R = 0$$

where ρ is the radius in cylinder coordinates (*ρ*^2^ = *x*^2^ + *y*^2^) and *S*, *P*, *Q*, and *R* are parameters derived from *d*, *h*, *b*, and *c* shown in Fig. 2a, respectively. In this paper, *d*, h/*d*, b/*d*, and c/*d* were set to 7.7 μm, 0.3542, 0.1752, and 0.6196, respectively, as suggested in ref. ^36^. We refer interested readers to previous studies^36,37^ for a more complete presentation of the DDA simulation of an RBC. By using a single simulated RBC, a cluster consisting of 15 identical RBCs was generated, as shown in Fig. 2b. In addition, we generated a synthetic cell phantom with four different RI values corresponding to the cytoplasm, nucleus, nucleolus, and lipids^32^. To derive the scattered field from the cluster and the cell phantom, samples were scanned by using 40 uniformly distributed illumination angles on a circle with an incident angle of 45°. For every simulation mentioned above, the sample with an RI of *n* was immersed in air, and the wavelength used was 600 nm. This is equivalent to a case in which the RI of the medium is *n*_0_ and the sample with an RI of *n* × *n*_0_ is illuminated at a wavelength of 600 × *n*_0_ nm. The number of dipoles per wavelength for both simulations was set to 12. Table 1 summarizes the numerical and experimental parameters used for the simulations as well as for the experiments.

**Table 1:** Reconstruction parameters

	Size	μm/pixel	n	*γ*	*C*	Iterations	Time/iteration (s)	
							LT-BPM	LT-SSNP
Cylinders	1024 × 256	0.15	1.05	1e–3	90	400	2.81	7.19
RBCs	512 × 512 × 180	0.15	1.05	1e–3	57	200	13.6	19.27
Cell phantom	350 × 350 × 128	0.15	1.0248 (cytoplasm), 1.0210 (nucleus), 1.0413 (nucleolus), and 1.0886 (lipids)	1e–3	57	200	3.45	5.01
Yeast	150 × 150 × 80	0.1	NA	0.25e–3	163	200	0.35	0.5
HCT116	256 × 256 × 170	0.1	NA	0.25e–3	160	200	3.14	4.36

### Experiments

The experiments were performed by using a conventional optical diffraction tomography configuration in which a spatial light modulator was used to control the illumination angle. A total of 360 holograms were recorded for each sample in a circular pattern with 1° resolution at an incidence angle of 35°. Additional details about the optical setup and sample preparation are provided in the supplementary section.

### Semisynthetic simulation

The semisynthetic measurements were calculated by using the reconstruction results acquired from the LT-BMP and the LT-SSNP as samples for the DDA. The size of the dipole was set to $\frac{\lambda }{{12{{n}}_0}} = 0.033\,{\mathrm{nm}}$, where *λ* = 0.532 nm is the wavelength of the laser and *n*_0_ = 1.338. Both the values were set from the values used in the experiments. The grid size of the reconstructions from the LT-BPM and the LT-SSNP was 99 nm. The reconstruction results were interpolated to a grid, one pixel of which was the size of a dipole. Then, we quantized the RI values by using the following equation: ${\mathrm{round}}(\frac{{n_{{\mathrm{recon}}}}}{{n_0}}\times1000)/1000$, where *n*_recon_ denotes the reconstructed RI values. Simulations were performed for 160 nonoverlapping angles, which were calculated from the experiments.

### Reconstruction algorithm

We implemented the algorithms by using custom scripts in MATLAB R2018a (MathWorks Inc., Natick, MA, USA) on a desktop computer (Intel Core i7-6700 CPU, 3.4 GHz, 32 GB of RAM). To accelerate the computation, a graphic-processing unit (GPU, GeForce GTX 1070) with custom-made functions based on the compute unified device architecture (CUDA) was utilized. The gradient, calculated from a data fidelity term, D($\bf{x}$), was ∂D($\bf{x}$)/∂$\bf{x}$, the amplitude of which is proportional to the amplitude of D($\bf{x}$). The LT-BPM and the LT-SSNP use different data fidelity terms. The LT-BPM calculates the difference in the fields *u*(*x,y,z*). In contrast, the LT-SSNP requires differences in both *u*(*x,y,z*) and its derivative *du*(*x,y,z*)/*dz*. Therefore, calibration of the optimization parameters between the methods is necessary to make the LT-BPM and the LT-SSNP use similar optimization parameters. The FISTA requires two parameters: step size (*γ*) and regularization parameter (*τ*). The calibration of those parameters can be performed by calculating the ratio *C* between $||u\left( {x,y,z} \right)||_2^2$ and $||u\left( {x,y,z} \right) + du\left( {x,y,z} \right)/dz||_2^2$. We approximated this as the average value of (1+1*ik*_z_)^2^ for the illumination *k*_*z*_s, which corresponds to a case in which *u*(*x,y,z*) is replaced with a planar wave, $e^{1i(k_xx + k_yy + k_zz)}$. Therefore, the LT-BPM, which uses the parameters *γ* and *τ*, can be directly compared with the LT-SSNP, which uses the parameters *γ*/*C* and *τ* × *C*. For convenience, we labeled the figures according to the parameters used for the LT-BPM. The actual parameter values for the LT-SSNP can be easily calculated given *C*, which is provided in Table 1. The total number of iterations used in the FISTA is also provided in Table 1. Twenty iterations were used for the TV optimization step in all cases.

### Overall scheme of the learning tomography

Both algorithms (LT-BPM and LT-SSNP) start from measured electric fields (including both amplitude and phase information) from the holographic data. An initial guess of the RI distributions is obtained by using the Rytov tomographic reconstruction method. By using either the BPM or the SSNP as the forward model, the scattered field is estimated given the plane-wave illumination propagating through this initial guess. The square of the difference between the estimated and the measured fields is the cost function, which is minimized by adjusting the index values contained in the forward model through the FISTA. At the same time, an intermediate step of regularizations such as smoothness and non-negativity is included. This process is repeated until the total cost function converges.

### Split-step non-paraxial method

In this section, we briefly describe the SSNP in 3D^23,24^, which is the physical forward model used in the LT-SSNP. Bhattacharya and Sharma^38^ implemented this method by using a matrix formalism for wave propagation in 3D. Here, we describe a fast Fourier transform implementation for more efficient use of memory.

The propagation of a scalar wave *u*(*x,y,z*) through a medium *n*(*x,y,z*) in 3D must satisfy the following wave equation:

4 $$\left( {\frac{{\partial ^2}}{{\partial x^2}} + \frac{{\partial ^2}}{{\partial y^2}} + \frac{{\partial ^2}}{{\partial z^2}}} \right)u\left( {x,y,z} \right) + k_0^2n^2\left( {x,y,z} \right)u\left( {x,y,z} \right) = 0$$

where *k*_0_ = 2*π/λ* is the free-space wavenumber for a given wavelength *λ* in a vacuum. Eq. (4) can be written in matrix form

5 $$\frac{\mathrm{d}{{\mathbf{v}}(x,y,z)}}{\mathrm{dz}} = {\mathbf{H}}(x,y,z){\mathbf{v}}(x,y,z)$$

where,

6 $${\mathbf{v}}(x,y,z) = \left[ {\begin{array}{*{20}{c}} {u(x,y,z)} \\ {\frac{{u(x,y,z)}}{{\partial z}}} \end{array}} \right]$$

and

7 $${\mathbf{H}}(x,y,z) = \left[ {\begin{array}{*{20}{c}} 0 & 1 \\ { - \left( {\frac{{\partial ^2}}{{\partial x^2}} + \frac{{\partial ^2}}{{\partial y^2}} + k_0^2n^2(x,y,z)} \right)} & 0 \end{array}} \right]$$

When we consider an inhomogeneous sample immersed in a homogeneous medium, *n*_0_, it is possible to split the matrix **H** into two terms that correspond to diffraction and phase modulation. Note that no approximation is assumed up to this point. We refer interested readers to the supplementary section for a detailed explanation.

## Supplementary information

Supplementary Information.
