# Supplementary material for: High-fidelity optical diffraction tomography of multiple scattering samples
Source: Light Sci Appl. 2019 Sep 11;8:82. doi: 10.1038/s41377-019-0195-1 (PMC6804780; doi:10.1038/s41377-019-0195-1)
Supplement: Supplementary file 1 — Supplementary Information. [file 41377_2019_195_MOESM1_ESM.pdf]

Supplementary Information for

High fidelity optical diffraction tomography of multiple scattering samples

Joowon Lim,* Ahmed B. Ayoub, Elizabeth E. Antoine and Demetri Psaltis

Ecole Polytechnique Fédérale de Lausanne, Optics Laboratory, CH-1015 Lausanne, Switzerland

**Corresponding author: joowon.lim@epfl.ch*

Scattering analysis in multiple cylinders

When there are multiple samples, it is very critical to model distortions in the wave front (phase modulation) introduced by the precedent samples which determine the illumination on subsequent samples. We generate synthetic measurements using Mie theory for 1-layer, 2-layers and 3-layers of cylinders as shown in Fig. S1a. Each layer contains 3 cylinders and light propagates along the z-axis and illumination angle varies from -45 degrees to +45 degrees in the xz plane. We denote the total electric field and incident field as u_{i_f} and $u_{i\_f}^{(in)}$, respectively, where i is the number of layers in the sample and f is the location of the imaging plane. (For example, u_{3_2} means the total electric field for the 3-layers sample and the image plane is at the center of the 2nd layer of cylinders.). Fig. S1b demonstrates the error introduced by the Rytov approximation by presenting the phase contrast between the total electric field and the incident field for each sample: angle$(u_{i_i}/u_{i_i}^{(in)})$ when $i = 1,2,3$. For the single layer case, we can fairly retrieve the information about the sample from the measured fields but it becomes harder as the number of layers increases. On the other hand, we can think of the total electric field from the 1-layer sample as the incident field on the second layer of the 2-layers sample. Therefore, the corrected phase, angle(u_{2_2}/u_{1_2}), preserves more information about the sample than the direct phase contrast, angle$(u_{2_2}/u_{2_2}^{(in)})$ as shown in Fig. S1c, which is also the case for the 3-layers sample. Therefore, taking into account the wave front distortions introduced by the precedent samples is important to achieve full 3D imaging and LT-SSNP gives significant improvements over the conventional Rytov approximation.

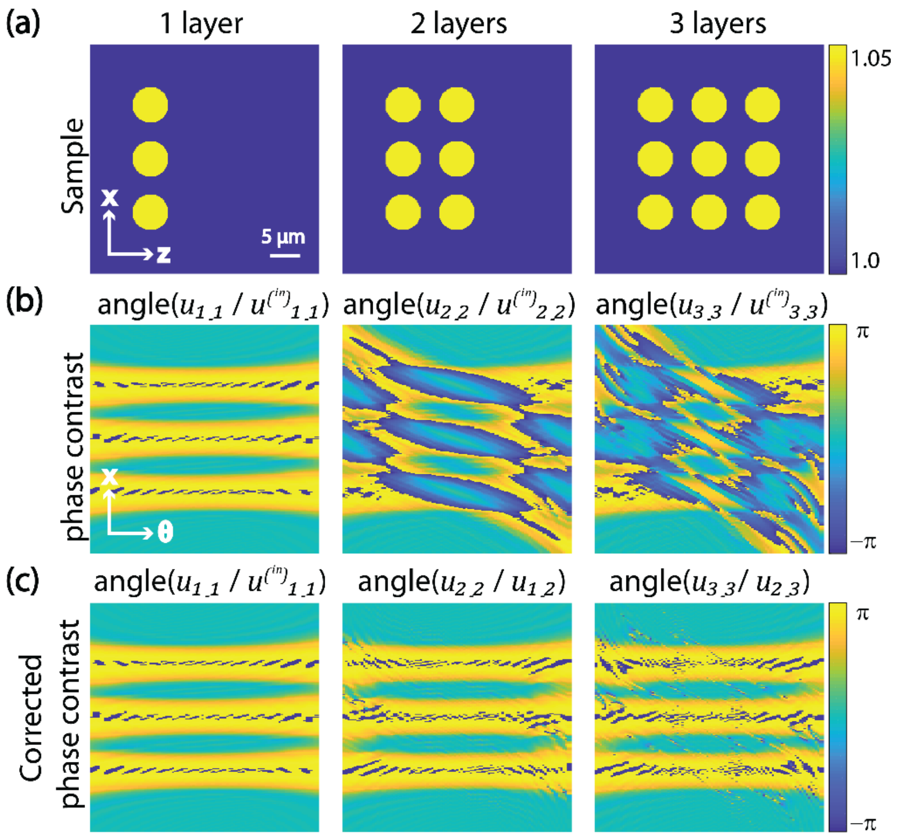

Fig. S1. Mie simulations for multiple cylinders. (a) 3 different samples. (b) phase contrasts for each sample. (c) corrected phase contrasts for each sample.

Data compression demonstrated on cell phantom using discrete dipole approximation

In this section, we compare various reconstruction algorithms from compressed measurements of the cell phantom generated using DDA. Reconstructions were performed using Rytov, linear tomography[1] and LT-SSNP using different numbers of projection angles (40, 20, 10 and 5) uniformly spaced in the range from 0 to 360 degrees as shown in Fig. S2. In the case of HCT116 cells, Rytov produces fairly good reconstructions with 360 full measurements, which means samples are not highly scattering. Therefore, combined with the single scattering forward model, linear tomography can handle fairly well data compression. However, in this case of the cell phantom, we can see that Rytov reconstructions are severely distorted and there is a limitation in reconstructing from down-sampled measurements even using the linear tomography. Unlike the linear tomography, LT-SSNP, which is the iterative scheme but with the accurate forward model, effectively handles the data compression producing consistent results with the ground truth.

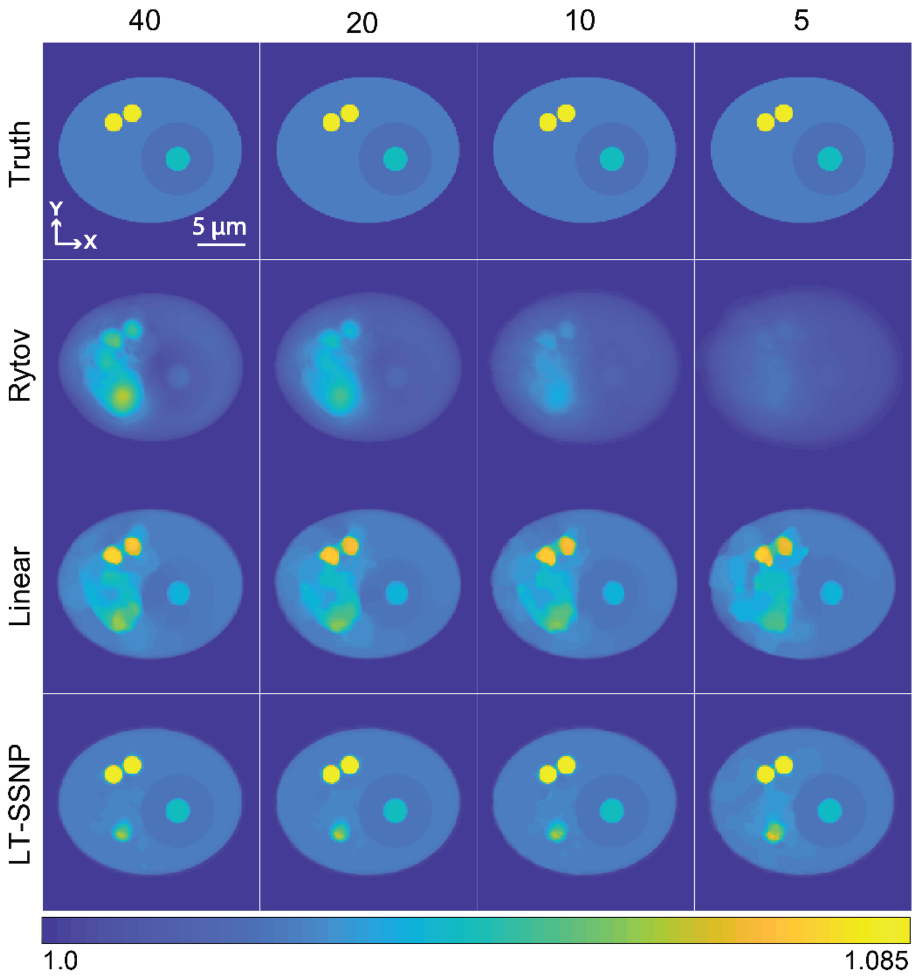

Fig. S2. Reconstruction results of the cell phantom using Rytov, linear tomography and LT-SSNP for down-sampled numbers of scanning angles.

Split-step non-paraxial method (SSNP)

In this section, we briefly describe SSNP in 3D[2,3] which is the physical forward model used in LT-SSNP. Bhattacharya and Sharma[4] implemented this method using a matrix formalism for wave propagation in 3D. Here, we describe a fast Fourier transform implementation for more efficient use of memory.

The propagation of a scalar wave $u(x,y,z)$, through a medium $n(x,y,z)$, in 3D can be described as

$$(\frac{\partial^2}{\partial x^2}+\frac{\partial^2}{\partial y^2}+\frac{\partial^2}{\partial z^2})u(x,y,z)+k_0^2n^2(x,y,z)u(x,y,z)=0, \tag{1}$$

where $k_0 = 2\pi/\lambda$ is the free space wavenumber for a given wavelength λ in vacuum. Eq. (1) can be written in matrix form:

$$\frac{d\mathbf{v}(x,y,z)}{dz}=\mathbf{H}(x,y,z)\mathbf{v}(x,y,z), \tag{2}$$

where

$$\mathbf{v}(x,y,z)=\begin{bmatrix} u(x,y,z) \\ \dfrac{u(x,y,z)}{\partial z} \end{bmatrix}, \tag{3}$$

and

$$\mathbf{H}(x,y,z)=\begin{bmatrix} 0 & 1 \\ -(\dfrac{\partial^2}{\partial x^2}+\dfrac{\partial^2}{\partial y^2}+k_0^2n^2(x,y,z)) & 0 \end{bmatrix}. \tag{4}$$

By applying the finite difference method for a small step, Δz to solve Eq. (2), we can get the following equation,

$$\mathbf{v}(x,y,z+\Delta z)=\exp(\mathbf{H}(x,y,z)\Delta z)\mathbf{v}(x,y,z). \tag{5}$$

When we consider an inhomogeneous sample immersed in a homogeneous medium, n_0, it is possible to split $\mathbf{H}$ into $\mathbf{H}_1$ and $\mathbf{H}_2$ which correspond to diffraction and phase modulation, respectively,

$$\mathbf{H}(x,y,z)=\mathbf{H}_1(x,y,z)+\mathbf{H}_2(x,y,z)=\begin{bmatrix} 0 & 1 \\ -(\dfrac{\partial^2}{\partial x^2}+\dfrac{\partial^2}{\partial y^2}+k_0^2n_0^2) & 0 \end{bmatrix}+\begin{bmatrix} 0 & 0 \\ k_0^2(n_0^2-n^2(x,y,z)) & 0 \end{bmatrix}. \tag{6}$$

Using Eq. (6), we can define $\mathbf{P}=\exp(\mathbf{H}_1\Delta z)$ and $\mathbf{Q}=\exp(\mathbf{H}_2\Delta z)$, then Eq. (5) can be split into two steps,

$$\mathbf{v}(x,y,z+\Delta z)=\mathbf{QPv}(x,y,z). \tag{7}$$

We omit the notation of (x,y,z) in $\mathbf{P}$, $\mathbf{H}_1$, $\mathbf{Q}$ and $\mathbf{H}_2$ for the sake of brevity. Using the Taylor series expansion, $\mathbf{P}$ can be written as

$$\mathbf{P}=\begin{bmatrix}\mathbf{I} & \mathbf{0}\\ \mathbf{0} & \mathbf{I}\end{bmatrix}+\mathbf{H}_1\Delta z+\frac{(\mathbf{H}_1\Delta z)^2}{2!}+\frac{(\mathbf{H}_1\Delta z)^3}{3!}+\ldots \tag{8}$$

Q can be simplified as follows:

$$\mathbf{Q}=\begin{bmatrix}\mathbf{I} & \mathbf{0}\\ \mathbf{0} & \mathbf{I}\end{bmatrix}+\mathbf{H}_2\Delta z, \tag{9}$$

since $(\mathbf{H}_2^m)$ is $\mathbf{0}$ when m is higher than 1.

Numerical implementation

We will denote the scalar field at the k-th slice by the vector $\mathbf{u}_k \in \mathbb{C}^M$ and the derivative of the field with respect to the optical axis, z, by the vector $\mathbf{u'}_{\mathrm{k}} \in \mathbb{C}^M$. The RI contrast of an inhomogeneous medium in 3D is represented by the vector $\mathbf{x}=\mathbf{n}-n_0 \in \mathbb{R}^N$ and $\mathbf{x}_{\mathrm{k}} \in \mathbb{R}^M$ denotes the k-th slice of $\mathbf{x}$. Derivative operations $\mathbf{H}_1$ in Eq. (6) can be implemented by taking the discrete Fourier transform (DFT) of the input signal, followed by Fourier kernel, and taking the inverse DFT again. Using Eq. (8) and Eq. (9), we can rewrite a discretized version of Eq. (7) for numerical implementation as follows:

$$\begin{bmatrix}\mathbf{u}_k\\ \mathbf{u}'_k\end{bmatrix}=\begin{bmatrix}\mathbf{I} & \mathbf{0}\\ \mathbf{G}_{21} & \mathbf{I}\end{bmatrix}\begin{bmatrix}\mathbf{K}_{11} & \mathbf{K}_{12}\\ \mathbf{K}_{21} & \mathbf{K}_{22}\end{bmatrix}\begin{bmatrix}\mathbf{u}_{k-1}\\ \mathbf{u}'_{k-1}\end{bmatrix}, \tag{10}$$

$$\begin{aligned}
\mathbf{G}_{21} &= \mathrm{diag}\{\mathbf{g}(\mathbf{x}_k)\}\\
\mathbf{K}_{11} &= \mathbf{F}^H[\mathbf{I}+\sum_{j=1}^{\infty}\mathrm{diag}(-\mathbf{s})^j\frac{\Delta z^{2j}}{(2j)!}]\mathbf{F}\\
\mathbf{K}_{12} &= \mathbf{F}^H[\mathbf{I}\Delta z+\sum_{j=1}^{\infty}\mathrm{diag}(-\mathbf{s})^j\frac{\Delta z^{2j+1}}{(2j+1)!}]\mathbf{F}\\
\mathbf{K}_{21} &= \mathbf{F}^H[\mathrm{diag}(-\mathbf{s})\mathbf{K}_{12}]\mathbf{F}\\
\mathbf{K}_{22} &= \mathbf{F}^H[\mathbf{K}_{11}]\mathbf{F}
\end{aligned} \tag{11}$$

where $g(\mathbf{x}_k)=-k_0^2(2n_0\mathbf{x}_k+\mathbf{x}_k{}^\circ\mathbf{x}_k)\Delta z$, ° signifies the Hadamard product. $\mathbf{F} \in \mathbb{C}^{M\times M}$ and $\mathbf{F}^H \in \mathbb{C}^{M\times M}$ are the DFT and inverse DFT, respectively. $\mathbf{s} \in \mathbb{C}^M$ denotes the Fourier kernel corresponding derivative operations, $-(\frac{\partial^2}{\partial x^2}+\frac{\partial^2}{\partial y^2}+k_0^2n_0^2)$, in Eq. (6): $\mathbf{s}=(k_0n_0)^2-\mathbf{k}_{\mathbf{x}}^2-\mathbf{k}_{\mathbf{y}}^2$ where $\mathbf{k}_{\mathbf{x}}$ and $\mathbf{k}_{\mathbf{y}}$ describe the Fourier components. For numerical stability, $\mathbf{s}$ components were multiplied with a mask which is 1 ($((k_0n_0*\sin(85^\circ))-\mathbf{k}_{\mathbf{x}}^{\mathbf{2}}-\mathbf{k}_{\mathbf{y}}^{\mathbf{2}}$>0) or 0 (otherwise). Note that the Eq. (10) consists of diffraction (DFT, Fourier kernel and inverse DFT) followed by phase modulation. For the sake of brevity, we can represent Eq. (10) as follows:

$$\mathbf{S}_k(\mathbf{x})=\mathbf{G}(\mathbf{x}_k)\mathbf{K}\mathbf{S}_{k-1}(\mathbf{x}), \tag{12}$$

where $\mathbf{S}_k(\mathbf{x}) = [\mathbf{u}_k^H, \mathbf{u}_k'^H]^H$, $\mathbf{G}(\mathbf{x}_k)$ and $\mathbf{K}$ correspond to the phase modulation and diffraction components, respectively.

Calculation of the gradient of SSNP

In previous work[5], it has been shown that the gradient of $\mathbf{D}^{(l)}(\mathbf{x})$ can be written as

$$[\nabla \mathbf{D}^{(l)}(\mathbf{x})] = [\frac{\partial \mathbf{D}^{(l)}(\mathbf{x})}{\partial x_1} \cdots \frac{\partial \mathbf{D}^{(l)}(\mathbf{x})}{\partial x_N}] = \mathrm{Re}\{(\mathbf{S}_K^{(l)}(\mathbf{x}) - \mathbf{y}_K^{(l)})^H [\frac{\partial}{\partial \mathbf{x}} \mathbf{S}_K^{(l)}(\mathbf{x})\}]\}. \tag{13}$$

In practice, we are interested in the column vector:

$$[\nabla \mathbf{D}^{(l)}(\mathbf{x})]^H = \mathrm{Re}\{[\frac{\partial}{\partial \mathbf{x}} \mathbf{S}_K^{(l)}(\mathbf{x})\}]^H (\mathbf{S}_K^{(l)}(\mathbf{x}) - \mathbf{y}_K^{(l)})\}. \tag{14}$$

Using Eq. (11) and Eq. (12), we can calculate the following:

$$\begin{bmatrix} \dfrac{\partial \mathbf{S}_k^{(l)}(\mathbf{x})}{\partial \mathbf{x}} \end{bmatrix} = \begin{bmatrix} \mathbf{K}_{11} & \mathbf{K}_{12} \\ \mathbf{K}_{21} + \mathbf{G}_{21}\mathbf{K}_{11} & \mathbf{K}_{22} + \mathbf{G}_{21}\mathbf{K}_{12} \end{bmatrix} \begin{bmatrix} \dfrac{\partial \mathbf{S}_{k-1}^{(l)}(\mathbf{x})}{\partial \mathbf{x}} \end{bmatrix} + \begin{bmatrix} \mathbf{0} \\ \mathrm{diag}\{(\mathbf{K}_{11}\mathbf{u}_{k-1} + \mathbf{K}_{12}\mathbf{u}'_{k-1})\}[\dfrac{\partial}{\partial \mathbf{x}} \mathbf{g}(\mathbf{x}_k)] \end{bmatrix} \tag{15}$$

Since $\frac{\partial \mathbf{S}_0^{(l)}(\mathbf{x})}{\partial \mathbf{x}} = 0$, it is possible to calculate the Hermitian of Eq. (16) by recursion. The process is in summarized in Algorithm 1.

Algorithm 1: Gradient calculation
Input: input field $\mathbf{y}_0$, output field $\mathbf{y}_K$, and current estimate of the RI distribution $\hat{\mathbf{x}}$.<br><br>Output: $[\nabla \mathbf{D}^{(l)}(\hat{\mathbf{x}})]^H$ the gradient.<br><br>Algorithm:<br><br>1) Compute the total field $\hat{\mathbf{y}} = \boldsymbol{S}(\hat{\mathbf{x}})$ using the SSNP recursion Eq. (12), keeping all the intermediate light-fields $\hat{\mathbf{y}}_k = \boldsymbol{S}_k(\hat{\mathbf{x}})$ in memory.<br>2) Compute the residual $\boldsymbol{r}_K = \hat{\mathbf{y}}_K - \mathbf{y}_K$ and set $\mathbf{s}_K = \mathbf{0}$.<br>3) Compute $\mathbf{s}_0 = [\frac{\partial}{\partial \mathbf{x}} \mathbf{S}_K^{(l)}(\mathbf{x})\}]^H \mathbf{r}_K$ using the following iterative procedure for $m = K, \ldots, 1$<br>a. $\mathbf{s}_{m-1} = \mathbf{s}_m + [\mathbf{0}, [\frac{\partial}{\partial \mathbf{x}} \mathbf{g}(\mathbf{x}_k)]^H]\ \mathrm{diag}\{\overline{[\mathbf{K}_{11}\ \mathbf{K}_{12}]\hat{\mathbf{y}}_{k-1}}\}\mathbf{r}_K$<br>b. $\mathbf{r}_{m-1} = \begin{bmatrix} \mathbf{K}_{11}^H & \mathbf{K}_{21}^H + \mathbf{K}_{11}^H\mathbf{G}_{21}^H \\ \mathbf{K}_{12}^H & \mathbf{K}_{22}^H + \mathbf{K}_{12}^H\mathbf{G}_{21}^H \end{bmatrix} \mathbf{r}_m$<br>4) Return $[\nabla \mathbf{D}^{(l)}(\hat{\mathbf{x}})]^H = Re\{\boldsymbol{s}_0\}$

We will briefly discuss reflections in SSNP. In reference[6], it has been shown that SSNP can be bidirectional and therefore it can simulate both forward and backward propagations simultaneously. In other words, $\mathbf{v}(x, y, z)$ contains both forward and backward fields without distinction. We consider an example in which a Gaussian beam illuminates a slab with a step change in RI (medium 1: 1, medium 2: 1.067) as shown in Fig. S3. In reality, the light field in medium 1 is the summation of the illuminating beam and its reflection at the interface, while medium 2 contains only the transmitted beam (Fig. S3a). Interestingly, when we simulate this experiment using SSNP without considering the reflection in medium 1, SSNP artificially introduces a computational artifact in medium 2 in order to satisfy conservation of energy (Fig. S3b). This artifact takes the form of a second beam of light propagating from medium 2 to medium 1 which destructively interferes with the reflections, canceling it out (Fig. S3c).

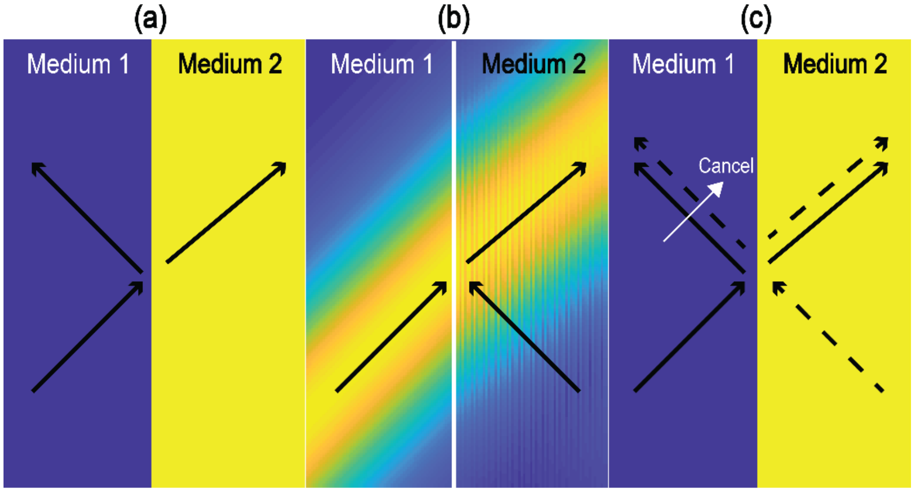

Fig. S3. Simulation of a Gaussian beam illuminating a slab (RI of medium 1: 1, RI of medium 2: 1.067) (a) True solution (b) SSNP solution (c) Interpretation of the SSNP artifact in (b) by introducing light going from medium 2 to medium 1 to cancel out the reflected light in medium 1.

Gradient filtering

We demonstrate the effect of this computational artifact on the gradient calculation using an example: a single 9 µm sphere (n: 1.067) immersed in air (n_0: 1) is illuminated by a planar wave propagating at a 45 degrees. The RI of the sphere is reconstructed using the Rytov approximation. The estimated field by SSNP at the detector plane contains both forward and backward propagations while measurements contain only forward propagations. Since the gradient is calculated by propagating the error between them, the resulting gradient produces two different gradients as shown in Fig. S4a; One is from the error between the forward propagation of SSNP and measurement, the other is from the error between the backward propagation of SSNP and measurement. The first goes through the sample but the later does not and it does not make sense to update the RI where no sample is present. The backward propagation is only a computational artifact of the SSNP method. Therefore, after calculating the gradient as described in Algorithm 1, we apply a Fourier transform followed by low-pass filtering to extract the gradient coming from the residual of transmitted fields as shown in Fig. S4b (The gradient after high-pass filtering is shown in Fig. S4c as reference). We use the low-pass filtered gradient for reconstruction. Due to the absence of reflected light on the illumination side, the

reconstructed RI distribution will be slightly underestimated. Therefore, we adjust the reconstruction, $\mathbf{x}_{recon}$ by dividing it by the transmittance with respect to the RI of the medium, n_0:

$$1-\frac{(\mathbf{x}_{recon}+n_0)-n_0}{(\mathbf{x}_{recon}+n_0)+n_0}=1-\frac{\mathbf{x}_{recon}}{\mathbf{x}_{recon}+2n_0}. \tag{16}$$

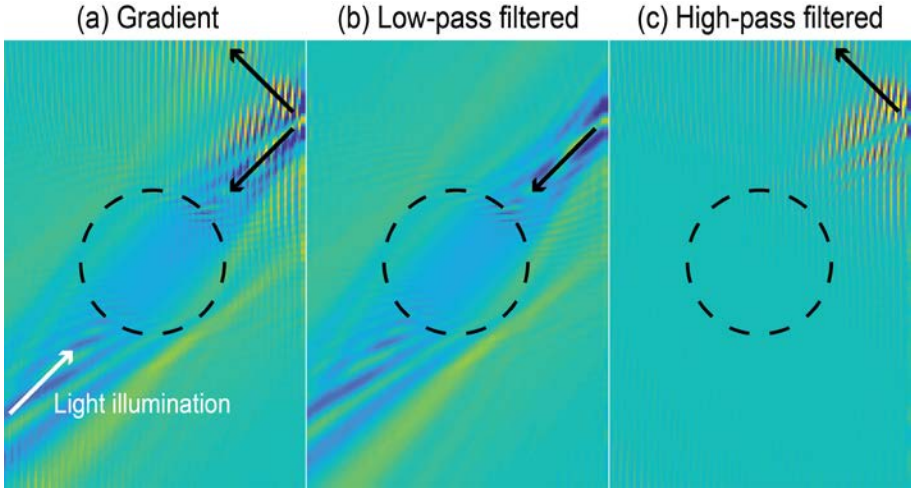

Fig. S4. Gradient filtering demonstration using an example; Rytov of a single 9 μm sphere (n=1.067) immersed in air (n_0=1) is illuminated by a planar wave propagating at a 45° angle of incidence. (a) Gradient calculated from residual containing both forward and backward propagating lights using Algorithm 1. The gradient is low-pass and high-pass filtered resulting in (b) and (c). Dotted lines represent the location of ground truth.

Evaluation of forward models

In this section, we evaluate BPM and SSNP using Mie theory for three spheres. Each sphere is 15 μm in diameter but has a different RI contrast (1.033, 1.067 and 1.1 immersed in the air) resulting in π, 2π and 3π phase contrasts when illuminated with 600 nm wavelength in air. We vary the illumination angle from 0 degree to 45 degrees and calculate the error in total electric fields: $||u_{Mie}-u_{Fwd}||^2$ where u_{Mie} and u_{Fwd} represent total electric fields generated using Mie theory and the other forward models (BPM or SSNP). We briefly explain BPM for the sake of completeness of this section.

BPM describes light propagation through an inhomogeneous medium using the envelope of the electric field, $u(x,y,z)=A(x,y,z)e^{ikz}$ where k is the wavenumber in the background medium. BPM consists of two parts: non-paraxial diffraction and phase modulation as followings,

$$A(x,y,z+dz)=e^{ik_0\Delta n(x,y,z)dz/\cos\theta}\times FT^H\left[FT\{A(x,y,z)\}\times e^{-i\left\{(k_x^2+k_y^2)/(k+\sqrt{k^2-k_x^2+k_y^2})\right\}dz}\right] \tag{17}$$

where k_0 is the wavenumber in vacuum, k_x and k_y represents frequency components corresponding to x and y axes. Δn is the contrast between RI of the sample ($n(x,y,z)$) and RI of the medium (n_0), FT (FT^H) represents 2D Fourier transform (inverse 2D Fourier transform). The term $dz/cos\theta$ in the phase modulation step geometrically corrects for the optical path length for tilted illumination at angle θ. In this section, we

tested BPM twice; one with dz as the step in the phase modulation and the other with $dz/cos\theta$ to see the impact of correction using $cos\theta$.

As shown in Fig. S5, the accuracy of BPM (without and with the cosine factor) rapidly deteriorates as the illumination angle increases. By comparing BPM without/with the cosine factor, the factor improves the accuracy of model. However, the gap in accuracy between with and without the cosine factor at higher illuminations decreases as the RI contrast increases. It means that there is limitation in geometrically correcting the optical path using the cosine factor. By contrast, the accuracy of SSNP stays stable making SSNP more accurate than BPM at high illumination angles. High illumination angles are critical in ODT to achieve good resolution in the optical axis. As mentioned in the previous section, SSNP is bidirectional and it simulates both forward and backward propagations simultaneously. We filtered the backward propagations of SSNP since we do not use the backward propagations in the iterative scheme by the gradient filtering [7]. In addition, since we divide the final reconstruction by reflectance in LT-SSNP, we calculate the reflectance, $\mathrm{R} = 1 - (\mathrm{x}/(\mathrm{x} + 2\mathrm{n}_0))$ where x is the RI contrast used for Mie simulation and n_0 is the medium RI. Then, x values multiplied by the reflectance serve as samples in SSNP simulations. The multiplication of the reflectance is introduced to compensate the missing signals to backward propagations which comes from the bidirectional property of SSNP.

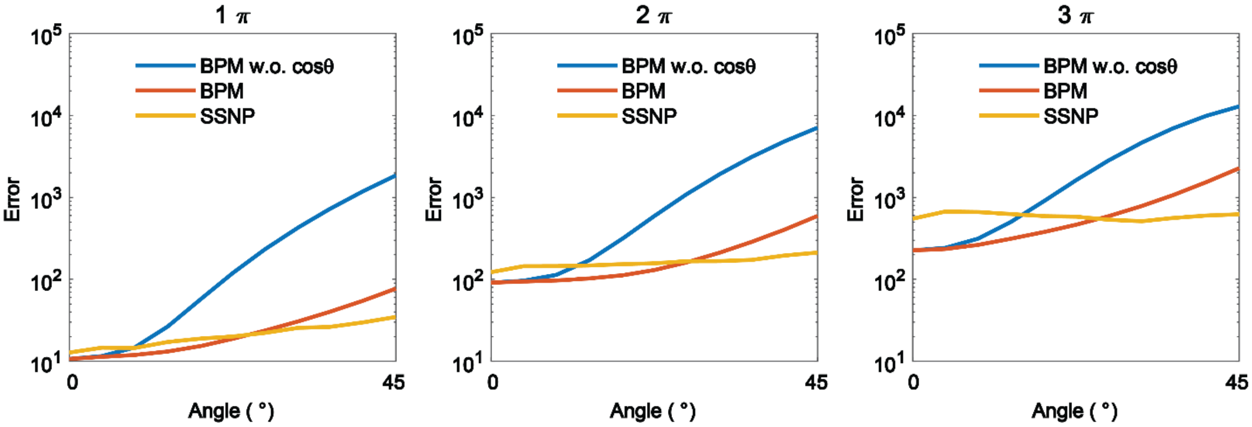

Fig. S5. Evaluation of total electric fields generated by BPM and SSNP using Mie theory as the ground truth for three spheres which differ in RI contrast values. The resulting phase delay for each sphere is written on each plot. The illumination angle for each sphere is increased from 0 degree to 45 degrees.

Experimental Setup

The optical system shown below used to acquire the data shown in this study used a diode pumped solid state 532 nm laser. The laser beam was first spatially filtered using a pinhole spatial filter. A beamsplitter was used to separate the input beam into a sample beam and a reference beam. The sample beam was directed onto the sample at different angles of incidence using a reflective LCOS spatial light modulator (SLM) (Holoeye) with a pixel size of 8 μm and resolution of 1080 x 1920 pixels. Different illumination angles were obtained by projecting blazed gratings on the SLM. In the experiments presented here, a blazed grating with a period of 25 pixels (200 μm) was circularly rotated with a resolution of 1 projection per degree for total projections. Two 4f

systems between the SLM and the sample permitted filtering of higher orders reflected from the SLM (due to limited fill factor and efficiency of the device) as well as magnification of the SLM projections onto the sample. Using a 100X oil immersion objective lens with NA 1.4 (Olympus), the incident angle on the sample corresponding to the 200 μm grating was 35°. The magnification of the illumination side was defined by the 4f systems we used before the sample. A third 4f system after the sample includes a 100X oil immersion objective lens with NA 1.45 (Olympus). The sample and reference beams were collected on a second beamsplitter and projected onto a scientific CMOS camera (Neo, Andor) with a pixel size of 6.5 μm and resolution of 2150 x 2650 pixels.

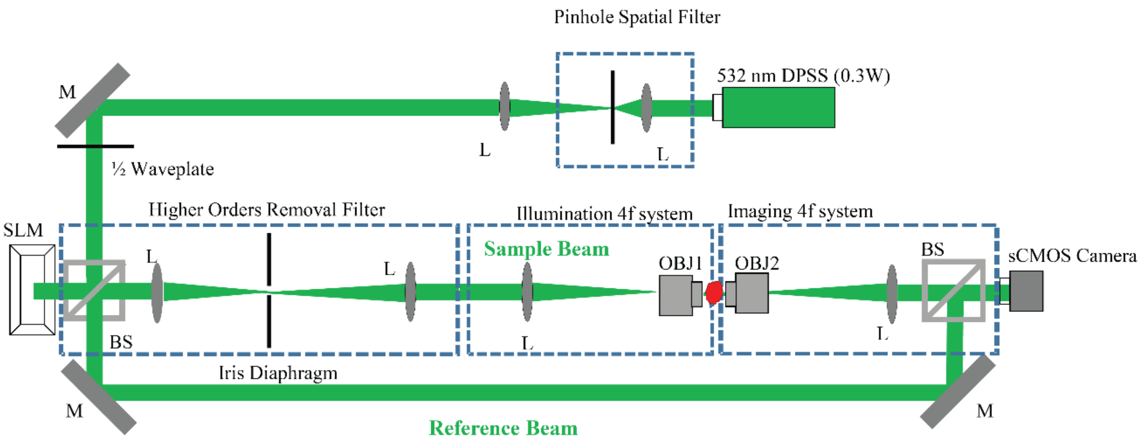

Fig. S6. Schematic for the experimental setup (M: Mirror, L:Lens, OBJ: Objective lens, BS: Beam splitter).

Sample preparation

Yeast cells were grown in a solution of agar with a refractive index of 1.338 (a change of 0.00125 for 0.5% increase in the Agar concentration). 2 g pure agar powder (AppliChem) was dissolved in 100 mL water, heated to 80C, and subsequently cooled, all while stirring with a magnetic stirrer. Once the solution reached 40C, 2 g fresh grocer's yeast was added to the solution. The yeast-agar solution was immediately pipetted onto a #1 coverslip and covered with a second coverslip and finally allowed to cool to room temperature for complete gel formation.

HCT-116 human colon cancer cells were cultured in McCoy 5A growth medium (Gibco) supplemented with 10% fetal bovine serum (Gibco). #1 coverslips were treated with a 5 μg/mL solution of fibronectin (Sigma) in phosphate-buffered saline (PBS) and air-dried at room temperature. Cells at passage 11 were detached from culture flasks using trypsin, seeded directly onto the fibronectin-treated coverslips, and incubated 24 hours in a 37C/5% CO_2 atmosphere until cells adhered and spread on the coverslips. Each sample was fixed for 10 minutes at room temperature in 4% paraformaldehyde in PBS, rinsed twice with PBS, and sealed with a second coverslip.

References

1 Lim, J., Goy, A., Shoreh, M. H., Unser, M. & Psaltis, D. Learning Tomography Assessed Using Mie Theory. *Physical Review Applied* **9**, 034027 (2018).

2 Sharma, A. & Agrawal, A. New method for nonparaxial beam propagation. *JOSA A* **21**, 1082-1087 (2004).

3 Sharma, A. & Agrawal, A. Non-paraxial split-step finite-difference method for beam propagation. *Optical and quantum electronics* **38**, 19-34 (2006).

4 Bhattacharya, D. & Sharma, A. Split step non-paraxial finite difference method for 3D scalar wave propagation. *Optical and Quantum Electronics* **39**, 865-876 (2007).

5 Kamilov, U. S. *et al.* Optical tomographic image reconstruction based on beam propagation and sparse regularization. *IEEE Transactions on Computational Imaging* **2**, 59-70 (2016).

6 Bhattacharya, D. & Sharma, A. Modelling of Optical Reflection Using a Nonparaxial Split-Step Finite Difference Beam Propagation Method. (2011).

7 Bhattacharya, D. & Sharma, A. Modeling of optical reflection using a nonparaxial split-step finite difference beam propagation method. *Proc. Indian National Science Academy* **77**, 193-205 (2011).
